# Engineering anisotropic electrodynamics at the graphene/CrSBr interface

**DOI:** 10.1038/s41467-025-56804-y

**Published:** 2025-02-21

**Authors:** Daniel J. Rizzo, Eric Seewald, Fangzhou Zhao, Jordan Cox, Kaichen Xie, Rocco A. Vitalone, Francesco L. Ruta, Daniel G. Chica, Yinming Shao, Sara Shabani, Evan J. Telford, Matthew C. Strasbourg, Thomas P. Darlington, Suheng Xu, Siyuan Qiu, Aravind Devarakonda, Takashi Taniguchi, Kenji Watanabe, Xiaoyang Zhu, P. James Schuck, Cory R. Dean, Xavier Roy, Andrew J. Millis, Ting Cao, Angel Rubio, Abhay N. Pasupathy, D. N. Basov

**Affiliations:** 1 Department of Physics, Columbia University, New York, NY USA; 2 Theory Department, Max Planck Institute for Structure and Dynamics of Matter and Center for Free-Electron Laser Science, Hamburg, Germany; 3 Department of Chemistry, Columbia University, New York, NY USA; 4 Department of Materials Science and Engineering, University of Washington, Seattle, WA USA; 5 Department of Applied Physics and Applied Mathematics, Columbia University, New York, NY USA; 6 Department of Physics, Pennsylvania State University, University Park, PA USA; 7 Department of Mechanical Engineering, Columbia University, New York, NY USA; 8 Research Center for Materials Nanoarchitectonics, National Institute for Materials Science, 1-1 Namiki, Tsukuba, Japan; 9 Research Center for Electronic and Optical Materials, National Institute for Materials Science, 1-1 Namiki, Tsukuba, Japan; 10 Center for Computational Quantum Physics, Flatiron Institute, New York, New York, USA; 11 Nano-Bio Spectroscopy Group, Universidad del País Vasco UPV/EHU, San Sebastián, Spain

**Keywords:** Nanophotonics and plasmonics, Two-dimensional materials, Optical properties and devices, Surfaces, interfaces and thin films, Optical properties and devices

## Abstract

Graphene is a privileged 2D platform for hosting confined light-matter excitations known as surface plasmon polaritons (SPPs), as it possesses low intrinsic losses and a high degree of optical confinement. However, the isotropic nature of graphene limits its ability to guide and focus SPPs, making it less suitable than anisotropic elliptical and hyperbolic materials for polaritonic lensing and canalization. Here, we present graphene/CrSBr as an engineered 2D interface that hosts highly anisotropic SPP propagation across mid-infrared and terahertz energies. Using scanning tunneling microscopy, scattering-type scanning near-field optical microscopy, and first-principles calculations, we demonstrate mutual doping in excess of 10^13 ^cm^–2^ holes/electrons between the interfacial layers of graphene/CrSBr. SPPs in graphene activated by charge transfer interact with charge-induced electronic anisotropy in the interfacial doped CrSBr, leading to preferential SPP propagation along the quasi-1D chains that compose each CrSBr layer. This multifaceted proximity effect both creates SPPs and endows them with anisotropic propagation lengths that differ by an order-of-magnitude between the in-plane crystallographic axes of CrSBr.

## Abstract (web summary)

The authors demonstrate a graphene/CrSBr heterostructure exhibiting anisotropic surface plasmon polariton (SPP) propagation in the mid-infrared and terahertz range. Charge transfer at the interface directs SPPs along the quasi-1D chains that compose each CrSBr layer, with propagation lengths varying by an order of magnitude between the two in-plane crystallographic axes.

## Introduction

Two-dimensional (2D) van der Waals (vdW) materials are ideal atomic-scale media for generating confined light spanning terahertz (THz)^1–4^, mid-^5–22^ /near-infrared^23^ (MIR/NIR) and visible^24–26^ energies. These materials possess phononic^9–16^, electronic^1–3,5–8,21–23^, or excitonic^24–26^ properties that cause the permittivity to become negative, providing the necessary conditions for hosting confined light-matter excitations known as polaritons. In general, 2D materials can support enhanced optical confinement, high electronic and dielectric tunability, and low losses, leading to the realization of polaritons displaying ballistic propagation^5^, in-plane^13–16,24^ and out-of-plane hyperbolicity^10,12^, electronically-tunable topology^9,17^, and canalization^9,13^ Among these materials, only graphene can host high-quality photonic propagation in the monolayer limit due to the uniquely high mobility of Dirac quasiparticles. Indeed, quality factors of 25 or greater have been observed for graphene surface plasmon polaritons (SPPs)^5,6^ at room temperature and 150 or greater at low temperature^5^. Conversely, other vdW media typically require in excess of ~10^2^ layers to achieve similar propagation lengths^10^. On the other hand, the isotropic nature of graphene limits its potential utility for lateral confinement and channeling of photonic modes – properties that have been observed in bulk slabs of intrinsically anisotropic materials such as V_2_O_5_^27^, α-MoO_3_^13–17^ and CrSBr^24^. The ability to impose uniaxial photonic properties on pristine sheets of graphene – free of nano-structuring and postprocessing – would enable in-plane manipulation and confinement of light propagating in an atomically-thin material.

Heterostructuring has recently been demonstrated as a viable route toward tuning the behavior of polaritons in vdW materials^11,17,20–22,28^. Here, optically-active modes in adjoining layers couple to 2D polaritons and create hybrid modes^17–19,29^. In addition, emergent phenomena arising from interfacial charge transfer can significantly influence photonic behavior in 2D heterostructures. Specifically, charge-transfer heterostructures (CTHs) enable non-volatile generation of SPPs in graphene^17,20–22^, and spatially-tunable losses of phonon-polaritons (PhPs) in hexagonal boron nitride (*h*BN)^11^. CTHs also allow nanometer-scale control of local conductivity, and thus support plasmonic cavities^20,30^, edge plasmon polaritons^22^, and plasmonic point-scatters^21,22^. Therefore, interfacing graphene with a 2D material possessing a different work function and optical anisotropy would leverage the combined effects of charge-transfer and optical coupling to control the directionality of graphene SPPs.

**Fig. 1:**
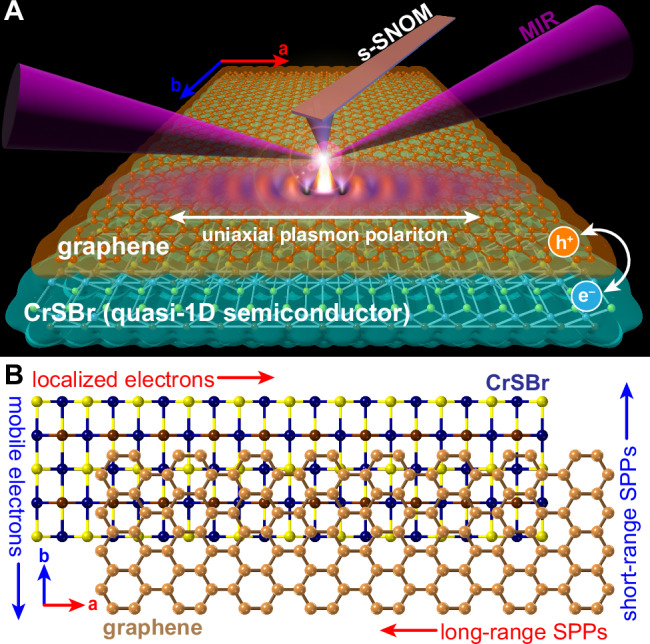
Schematic of charge transfer and uniaxial plasmon polariton propagation in graphene/CrSBr heterostructures. **A** Overview of s-SNOM measurements in graphene/CrSBr heterostructures. The difference in work functions between these layers leads to hole-doped graphene and electron-doped CrSBr. Uniaxial surface plasmon polaritons (SPPs) are generated upon illumination of the AFM tip with mid-infrared (MIR) light. **B** CrSBr displays a highly anisotropic crystal structure, forming 1D chains along the in-plane *a*-axis. The resulting electronic structure is quasi-1D, with *b*-axis carriers possessing a much higher mobility than *a*-axis carriers. Proximate interactions between graphene SPPs and electron-hole excitations in the underlying CrSBr leads to preferential SPP propagation along the 1D *a*-axis chains.

In this study, we demonstrate the ability to create uniaxial SPPs in graphene through heterostructuring with the air-stable vdW magnet CrSBr (Fig. 1A). CrSBr possesses significant in-plane structural anisotropy (Fig. 1B) that is reflected in its electronic and optical properties^24^. Each CrSBr layer is composed of 1D chains that result in a quasi-1D electronic structure^31–34^, where the CrSBr *a*-axis hosts flat conduction bands (CBs) that suppress efficient electrical conductivity while the *b*-axis CBs are more dispersive and are thus electrically conductive. Using a combination of scattering-type scanning near-field optical microscopy (s-SNOM) and scanning tunneling microscopy and spectroscopy (STM/STS), we visualize emergent electronic and nano-optical behavior arising from interfacial charge transfer and electronic anisotropy at the graphene/CrSBr interface. Our STM/STS results provide strong evidence of >0.5 eV shift in the Dirac-point energy (*E*_Dirac_) of graphene, indicating significant charge transfer with CrSBr (*n* > 10^13 ^cm^–2^) and confirming a theoretical prediction^35^. STM data also reveal topographic and density-of-states (DOS) features associated with a second-order moiré pattern, exhibiting a long-range, atomically clean interface.

In additional, we used s-SNOM to visualize the charge transfer-enabled SPPs in graphene/CrSBr. The dispersive behavior of these SPPs quantifies the magnitude of interfacial charge transfer consistent with the STS data. Our s-SNOM data further reveal a roughly order-of-magnitude difference in the SPP propagation lengths at MIR frequencies between the two in-plane crystallographic axes of CrSBr, along with a systematic suppression of the *b-*axis SPP group velocity at THz frequencies. We find that this highly anisotropic SPP behavior arises due the exceptional enhancement of optical anisotropy within the electron-doped interfacial layer of CrSBr. Here, the doped CrSBr possesses anisotropic electron-hole excitations that impart direction-dependent damping on SPPs with respect to the CrSBr in-plane crystallographic axes (Fig. 1A, B). The totality of our analysis indicates that emergent intra- and interband transitions play a significant role in the observed SPP anisotropy. This interpretation is supported by first-principles density functional theory (DFT) calculations, and provides a novel route for in-plane manipulation of confined light in atomically-thin media.

## Results

### Atomically-resolved topography and electronic structure

**Fig. 2:**
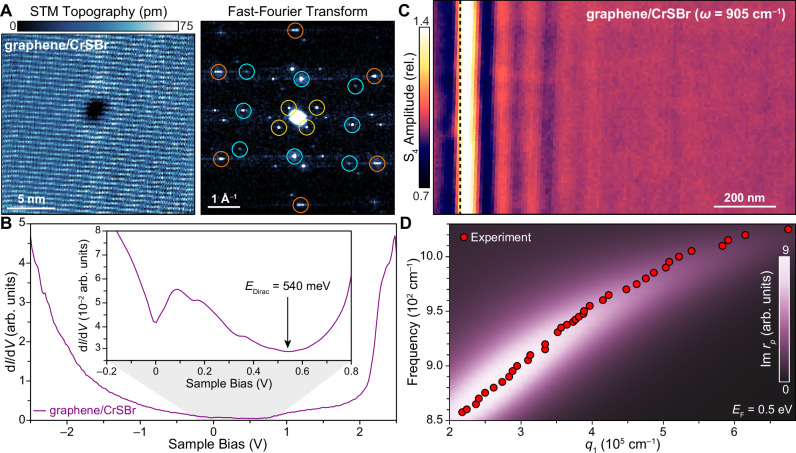
Multi-modal characterization of interfacial charge transfer in graphene/CrSBr heterostructures. **A** Left panel: Atomically-resolved topographic STM image of a graphene/CrSBr heterostructure (*V*_S_ = 1.2 V, *I* = 50 pA, *T* = 5.7 K). Right panel: Fast Fourier transform (FFT) of topographic data shows Bragg peaks associated with the graphene atomic lattice (orange circles), the CrSBr atomic lattice (cyan circles) and the second-order moiré pattern (yellow circles) corresponding to a twist angle of ~4° between the graphene armchair axis and CrSBr *a*-axis. **B** STS collected on a graphene/CrSBr heterostructure (*V*_S_ = 1.8 V, *I* = 50 pA). Inset: Low bias STS (*V*_S_ = 0.3 V, *I* = 100 pA) shows a d*I*/d*V* minimum at 540 mV corresponding to the Dirac-point energy (*E*_Dirac_) of graphene shifted due to interfacial charge transfer with the underlying CrSBr. Additional nearby d*I*/d*V* minima are also observed at 0 V, 150 mV, and 330 mV. **C** Typical s-SNOM image of a graphene/CrSBr heterostructure showing oscillations in the near-field S_4_ amplitude that are characteristic of SPPs (*ω* = 905 cm^–1^; S_4_ normalized relative to the value in the graphene/CrSBr bulk). The graphene is draped over the CrSBr edge (dashed black line), creating a sharp gradient in the graphene charge density that acts as a hard boundary for plasmonic reflections. **D** The experimental SPP dispersion for graphene/CrSBr (red circles) extracted from the line profiles of SPP fringes collected at different frequencies (see Fig. S3). Calculated Im *r*_p_ for the experimental stack using an input value of *E*_F_ = 0.5 eV for the graphene Fermi energy. Maxima in the Im *r*_p_ correspond well with the experimental dispersion, indicating that the SPP behavior is consistent with an 0.5 eV shift in *E*_Dirac_ of graphene due to charge transfer with the underlying CrSBr.

Figure 2A shows a characteristic STM topographic image of a graphene/CrSBr heterostructure. Short- and long-range modulations in the atomic-scale landscape are observed that resemble striped moiré patterns previously reported on heterostructures of graphene with anisotropic 2D materials^36–38^. A series of Bragg peaks visible in the fast Fourier transform (FFT) of STM topography (Fig. 2A; right panel) indicates the origin of these features. These include the graphene (orange circles) and CrSBr (cyan circles) atomic lattices, and a second-order moiré pattern (yellow circles) consistent with a 4° rotation of the CrSBr *a*-axis with respect to the graphene armchair axis. Our ability to simultaneously image the graphene and CrSBr atomic lattices along with the second-order moiré pattern demonstrates that graphene/CrSBr forms high-quality, atomically clean interfaces with minimal structural and twist disorder.

An STS spectrum averaged over the field of view in Fig. 2A is shown in Fig. 2B. The point spectrum shows local density of states (LDOS) features derived from both graphene and CrSBr. A wide spectral tail is observe spanning negative sample biases along with broad shoulders centered at 1.0 V and 2.5 V. We note that the spectra display intensity modulations likely due to moiré-induced variations in the local vacuum potential (Fig. S1). In addition, we can resolve anisotropic patterns in the vicinity of point defects, revealing electronic anisotropy that aligns with the crystallographic axes of CrSBr (Fig. S1). To isolate for LDOS features representative of graphene, we focus on sample biases spanning –0.2 to 0.8 V (Fig. 2B, inset). We observe a series of LDOS minima in the range 0 V to 0.54 V. Assigning *E*_Dirac_ to the global minimum in the STS spectrum suggests significant interfacial charge transfer corresponding to 0.54 eV hole-doping of graphene (the Dirac point is *E*_Dirac_ = 0.54 eV above the Fermi level), which gives a transferred charge density of $n=\frac{1}{\pi }{\left(\frac{{E}_{{Dirac}}}{\hslash {v}_{F}}\right)}^{2}=$ ~2 × 10^13 ^cm^–2^ according to the Dirac cone model (here the graphene Fermi velocity is *v*_F_ = 10^8 ^cm/s). Indeed, measurement of the CrSBr work function using KPFM yields a value of *W*_CrsBr_ ≈ 5 eV (Fig. S2), which is larger than that of graphene (*W*_graphene_ = 4.6 eV)^39^ and is consistent with interfacial hole doping of graphene and electron doping of CrSBr in graphene/CrSBr heterostructures.

### Characterization of the SPP dispersion

Interfacial charge transfer in graphene/CrSBr can be unambiguously quantified from s-SNOM measurements probing the frequency-dependent SPP behavior. Figure 2C shows a map of the near-field amplitude S_4_ at *ω* = 905 cm^–1^ collected on graphene draped over the edge of trilayer CrSBr (CrSBr edge indicated by black dashed line). While SPP fringes are typically observed near native graphene edges, the large difference in the graphene conductivity coinciding with the CrSBr edge generates a boundary capable of reflecting tip-launched SPPs and in-coupling free-space light in analogy to a true graphene edge. Thus, the characteristic fringe pattern in Fig. 2C is evidence of strong graphene-CrSBr interfacial charge transfer. We note that in Fig. 2C, the observed SPPs are propagating along the CrSBr *a*-axis.

To further quantify the behavior of graphene/CrSBr SPPs, we collect a series of images for frequencies spanning 850 to 1020 cm^–1^. Taking the SPP line profile at each frequency (Fig. S3), we can extract the complex-valued wavevector ($q={q}_{1}+i{q}_{2}$) using established fitting procedures accounting for both tip-launched and edge-launched SPPs^6,22^. In particular, the experimental SPP dispersion $\omega ({q}_{1})$ encodes the graphene charge carrier density and is plotted in Fig. 2D (red circles). We compare the experimental *a*-axis SPP dispersion to the calculated imaginary component of the *p*-polarized reflection coefficient, Im *r*_p_, whose maxima trace the expected SPP dispersion. To calculate Im *r*_p_, we input reported optical parameters^40,41^, our *a*-polarized far-field measurements of CrSBr (Fig. S4), and *E*_Dirac_ = 0.5 eV for graphene (as informed by our STS measurements) (Fig. 2D). The experimental dispersion aligns well with maxima in Im *r*_p_ despite there being no free parameters in our model. Thus, our near-field data validate the assignment of *E*_Dirac_ = 0.54 eV shown in Fig. 2B, confirming significant charge transfer in graphene/CrSBr heterostructures. We speculate that the additional LDOS minima observed in the 0 V to 0.54 V range in Fig. 2B arise due to one or a combination of (1) Van Hove singularities in the electron doped CrSBr layer, (2) inelastic tunneling^42^ and/or (3) superlattice Dirac points emerging from moiré-induced Brillouin zone folding^43,44^.

### Uniaxial SPPs

**Fig. 3:**
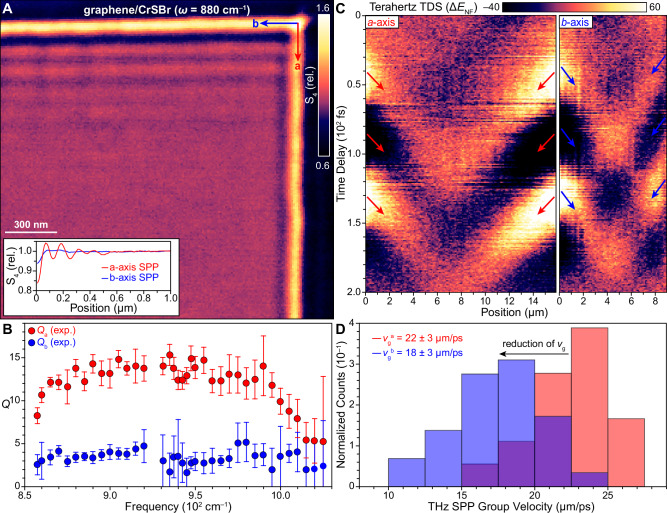
Proximity-induced anisotropy and uniaxial SPPs in graphene/CrSBr heterostructures. **A** Map of the near-field S_4_ amplitude in a graphene/CrSBr heterostructure showing multiple SPP fringes propagating along the CrSBr *a*-axis, while *b*-axis fringes are highly suppressed. Inset: The average line profiles of SPP fringes along the *a*-axis (red curve) versus the *b*-axis (blue curve) show a significantly diminished decay length for the latter (*ω* = 880 cm^–1^; S_4_ normalized relative to the value in the graphene/CrSBr bulk). **B** The experimentally-extracted frequency-dependent *Q*-factor for SPPs propagating along the *a*-axis (red circles) and *b*-axis (blue circles). Error bars are extracted from the standard error of ${q}_{1}$ and ${q}_{2}$ when fitting the line profiles in Fig. S3. **C** Left panel: Space-time map of the near-field plasmon electric field ($\Delta {E}_{{NF}}$) conducted along the *a*-axis of a graphene/CrSBr heterostructure (See Fig. S7 for background subtraction procedure). The edges of the images correspond to the edges of CrSBr. The red arrows indicate extrema that correspond to the worldlines of propagating SPPs. The group velocity of *a*-axis modes ${v}_{g}^{a}$ is twice the slope of the worldline. Right panel: The same as the left panel but for *b*-axis propagating modes with blue arrows indicating the SPP worldlines with group velocity ${v}_{g}^{b}$. **D** Histograms of the associated values of ${v}_{g}^{a}$ (red bars) and ${v}_{g}^{b}$ (blue bars) extracted from *N* ≥ 18 worldlines for each direction showing a systematic suppression of ${v}_{g}^{b}$ compared to ${v}_{g}^{a}$.

While s-SNOM measurements of *a*-axis propagating SPPs yield several observable fringes, we find that *b*-axis SPPs have a more subtle appearance. Figure 3A shows the corner of an exfoliated CrSBr microcrystal that is encapsulated with graphene. At this frequency (880 cm^–1^), at least five observable fringes emanate along the *a*-axis from the top edge, while SPP fringes are nearly unobservable along the *b*-axis. The inset of Fig. 3A shows the average SPP line profiles along both CrSBr crystallographic axes. One faint *b*-axis fringe is observable (blue curve), while the *a*-axis curve shows multiple significant oscillations (red curve). This behavior is observed at all experimental MIR frequencies (Fig. S3), indicating SPP damping with significant in-plane anisotropy. To quantify this anisotropy, we extract the SPP *Q*-factor ($Q=\frac{{q}_{1}}{{q}_{2}}$) from the experimental SPP fringe profile (Fig. 3B). We find that SPPs have significant additional losses along the *b*-axis compared to the *a*-axis (i.e., *Q*_a_ > *Q*_b_) with up to an order-of-magnitude difference in the associated *Q*-factors across the range *ω* = 850 – 1020 cm^–1^. Note that *Q*_a_ is comparable though smaller than nominal values obtained for SPPs measured under similar conditions in high quality *h*BN-encapsulated structures (*Q*_*h*BN_ ≈ 20)^6^, while *Q*_b_ is likewise significantly suppressed. We remark that the conductivity due to Dirac electrons of graphene is isotropic. It is therefore evident that the CrSBr in our structures is acting not only as a reservoir for interfacial charge doping but also imposes anisotropic damping on graphene SPPs in the MIR that depends on the underlying orientation of the CrSBr crystallographic axes. Notably, the observed difference in *Q*_a_ and *Q*_b_ is far in excess of what can be attributed to the intrinsic optical anisotropy of undoped CrSBr (Fig. S4), which shows almost no anisotropy (dashed curve in Fig. S3D), even though pristine CrSBr has strong anisotropy in its lattice.

### SPP anisotropy at THz energies

Nano-optical measurements at THz frequencies also reveal significant anisotropic SPP behavior. Figure 3C (left panel) shows a characteristic THz space-time map of the near-field SPP electric field ($\Delta {E}_{{NF}}$) ^45^ running parallel to the *a*-axis, with the CrSBr edges corresponding to the left and right edges of the panel. Extrema in the space-time map trace the SPP worldlines (red arrows in Fig. 3C, left panel) whose slope provide an explicit measure of the SPP group velocity, ${v}_{g}=2\frac{\Delta x}{\Delta t}$, as recently discovered^45^. Extracting the *a*-axis group velocity ${v}_{g}^{a}$ from our space-time maps yields an average value of 22 ± 3 μm/ps. Space-time maps collected along the *b*-axis also show SPP worldlines (Fig. 3C, right panel) whose associated group velocity ${v}_{g}^{b}$ is 18 ± 3 μm/ps. Histograms of the measured *a*- and *b*-axis group velocities can be found in Fig. 3D, revealing a systematic reduction of ${v}_{g}^{b}$ compared to ${v}_{g}^{a}$. Thus, our space-time maps demonstrate that proximity-induced suppression of *b*-axis SPP propagation extends from the MIR down to THz energies.

### Mechanism for Uniaxial SPPs

**Fig. 4:**
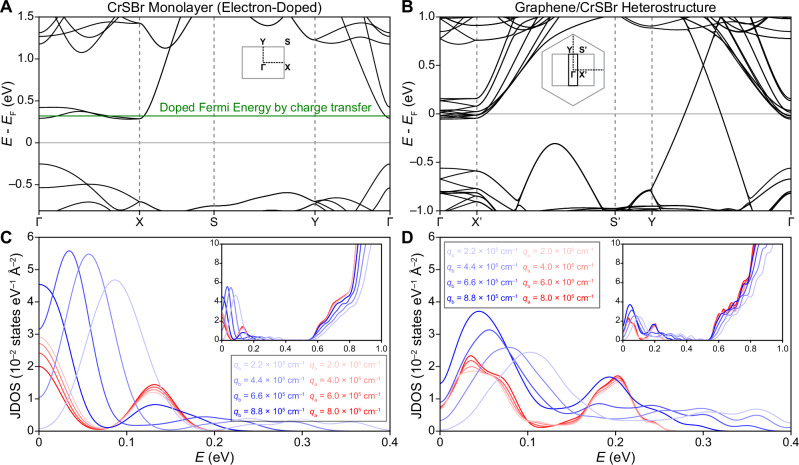
First-principles calculation of the JDOS for electron-doped CrSBr monolayer and graphene/monolayer CrSBr heterostructure. **A** Band structure of free-standing monolayer CrSBr calculated by DFT with PBE functional. The solid green line indicates the Fermi level of the CrSBr due to charge transfer with graphene (The charge transfer is ~0.03 electrons per CrSBr unit cell). The gray rectangle represents the first Brillouin zone of monolayer CrSBr with the high symmetry points indicated. **B** Band structure of graphene/monolayer CrSBr heterostructure calculated by DFT with PBE functional. The first Brillouin zones of graphene (gray hexagon), CrSBr (gray rectangle), and their heterostructure (black rectangle) are shown with the high symmetry points of the supercell indicated. The points X’ and S’ are the zone-folded analogs of the X and S points in (**A**). **C** The calculated joint density of state (JDOS) for an electron-doped CrSBr monolayer with the Fermi level marked by the solid green line in panel (**A**). The JDOS corresponds to the plasmon damping transition with momentum ${{{\bf{q}}}}$ and transition energy $E$ from the occupied and unoccupied manifolds, defined by ${{{\rm{JDOS}}}}\,\left(E,{{{\bf{q}}}}\right)=\int \delta \left(E-\left[{E}_{c}\left({{{\bf{k}}}}+{{{\bf{q}}}}\right)-{E}_{v}\left({{{\bf{k}}}}\right)\right]\right){d}^{3}{{{\bf{k}}}}$. Red curves denote the JDOS corresponding to the plasmon damping transition in the *a* direction ($|{{{\bf{q}}}}|={q}_{a}$), and blue curves the *b* direction ($|{{{\bf{q}}}}|={q}_{b}$). Curves with different hues denote different values of ${q}_{a}$ and ${q}_{b}$ as indicated in the plot legend. **D** The calculated JDOS for the graphene/monolayer CrSBr heterostructure. We note that transitions between band folded states do not contribute to the plasmon damping process. Therefore, the plotted quantity excludes transitions between zone-folded states. The insets in both (**C**) and (**D**) show the JDOS over a larger energy range. The anisotropy in JDOS is significant below ~0.4 eV, where the transitions are mostly from the flat bands near the Fermi level in the $a$ direction, and from the very dispersive bands near the Fermi level in the $b$ direction, respectively. At higher energy the JDOS is quite isotropic since the transitions involve the more isotropic band complexes above 1.0 eV and below −0.5 eV in panel (A).

In order to understand the microscopic origins of SPP anisotropy, we perform first-principles DFT calculations on model heterostructures consisting of graphene on monolayer CrSBr (Fig. 4B) and graphene on bilayer CrSBr (Fig. S5A–C). The monolayer-on-monolayer band structure is plotted in Fig. 4B (see Fig. S5 for monolayer-on-bilayer and spin-polarized band structures). Characteristic features of the isolated graphene and CrSBr layers can be clearly identified, such as the graphene Dirac cone and the quasi-1D structure of the CrSBr CB. Notably, the theoretical value of *E*_Dirac_ is ~0.50 eV (*n* = ~2 × 10^13 ^cm^–2^ within the Dirac cone model) indicating significant hole-doping of the graphene layer—in good agreement with both tunneling and nano-optical experiments (Fig. 2B, D), as well as previous theoretical predictions^35^. We note that most of the charge (>80% according to our calculations) transferred from graphene is found to be localized at the top-most CrSBr layer due to the formation of a potential gradient within CrSBr. In addition, the calculated *E*_Dirac_ = 0.5 eV in the graphene/bilayer CrSBr calculation (Fig. S5D) is mirrored in the graphene/monolayer CrSBr analysis (Fig. 4B), further indicating that the charge transfer is confined to the interfacial CrSBr layer. Indeed, other charge-transfer heterostructures are predicted to possess charge localization primarily in the interfacial layers due to similar effects^46^.

Our DFT calculations also show that the conduction band of CrSBr is now significantly electron doped and can thus support new intra- and interband transitions. Notably, free-carriers associated with the conduction band of doped CrSBr are highly anisotropic (*m*_a_ = *m*_Γ→Χ_ = 3.1*m*_e_ and *m*_b_ = *m*_Γ→Υ_ = 0.2*m*_e_) indicating that doped CrSBr is much more conductive along the *b*-axis than along the *a*-axis. Hence, the associated anisotropic Drude response of electron-doped CrSBr is likely to impart direction-dependence to both plasmonic group velocity and damping. In the case of interband transitions, the conduction band of doped CrSBr resides in close proximity to another unoccupied band (Fig. 4A) whose energy separation is anisotropic and overlaps with the SPP energies probed in our experiment. Thus, emergent interband transitions accessed through electron doping CrSBr will likely also induce anisotropy in the SPP response of graphene/CrSBr.

To explore the respective roles of intra- and interband transitions on the creation of uniaxial SPPs, we next investigate the origin of direction-dependent surface plasmon damping from first-principles calculations. We evaluate the overall plasmon scattering rate $\gamma$ using Fermi’s golden rule:

1 $$\gamma \left(E,{{{\bf{q}}}}\right)=\frac{2\pi }{{{\hslash }}}\int \delta (E-\left[{E}_{c}\left({{{\bf{k}}}}+{{{\bf{q}}}}\right)-{E}_{v}\left({{{\bf{k}}}}\right)\right]{\left|\left\langle {\psi }_{c,{{{\bf{k}}}}{{{\boldsymbol{+}}}}{{{\bf{q}}}}} | {H}_{{{\mathrm{int}}}} | {\psi }_{{{v}},{{{\bf{k}}}}}\right\rangle \right|}^{2}){d}^{3}{{{\bf{k}}}}$$

Where ${E}_{c}$ and ${E}_{v}$ are the energies of unoccupied and occupied states, respectively, **k** are the crystal momenta, **q** the SPP momenta, and ${H}_{{{\mathrm{int}}}}$ represents the electron-photon interaction Hamiltonian (e.g., an electric dipole interaction ${H}_{{{\mathrm{int}}}}=\frac{e}{{mc}}{{{\bf{A}}}}\cdot {{{\bf{p}}}}$, with ${{{\bf{A}}}}$ representing the vector potential of the incident field, and ${{{\bf{p}}}}=i\hslash \nabla$ representing the momentum operator). The form of $\gamma$ includes the joint density of states (JDOS $=\int \delta (E-[{E}_{c}({{{\bf{k}}}}+{{{\bf{q}}}})-{E}_{v} ({{{\bf{k}}}})]){d}^{3} {{\bf{k}}}\left)\right.$ where the integrand is modulated by the matrix elements of ${H}_{{{\mathrm{int}}}}$. We have checked that the latter matrix element varies by less than 10% for different ${k}_{x}$. This is because the wavefunction character along the $\Gamma$ to $X$ direction does not change significantly. Along the $\Gamma$ to $Y$ direction, only states close to the minima of the parabolic band are relevant to transitions that influence plasmon damping, and thus the matrix element stays approximately constant. Therefore, we use the JDOS rather than the full expression in Eq. 1 to quantify the anisotropic plasmon scattering rate by sampling electronic transitions whose crystal momenta match the momenta of the SPPs in the MIR ($\sim 2-8\times {10}^{5}$ cm^–1^). Here we consider two model structures: (1) free-standing monolayer CrSBr that has been electron-doped to match the charge transfer with graphene (Fig. 4A), and (2) a graphene/monolayer CrSBr heterostructure (Fig. 4B). By comparing the results of freestanding CrSBr and its heterostructure with graphene, we aim to parse the separate effects of charge doping and the presence of the graphene layer on the calculated scattering rate.

Along the *a*-axis, the calculated JDOS for the free-standing doped CrSBr shows two peaks at ~0.0 eV and ~0.14 eV (red curves, Fig. 4C) that mirror JDOS peaks in graphene/monolayer CrSBr at ~0.03 eV and ~0.20 eV (red curves, Fig. 4D). For both structures, the lower energy peaks mainly come from intraband transitions in the two closely packed flat bands near the Fermi level along $\varGamma -X$, while the higher energy peaks mainly come from interband transitions between these bands. We note that for graphene/monolayer CrSBr, there are also packed bands due to zone folding (Fig. 4B). Transitions between states where momentum conservation is only made possible due to zone folding will be improbable since the wavefunction overlap between initial and final states is small, and thus the matrix element $\left\langle {\psi }_{c,{{{\boldsymbol{k}}}}{{{\boldsymbol{+}}}}{{{\bf{q}}}}} | {H}_{{{\mathrm{int}}}} | {\psi }_{{{v}},{{{\bf{k}}}}}\right\rangle$ will be negligible. Therefore, transitions between zone-folded states are excluded from our calculation. The plasmon damping contribution from the graphene layer is modest compared to that of the CrSBr layer due to the large dispersion of the graphene layer, which is confirmed by the similarity between the two results shown in Fig. 4C, D. Notably, both model structures show a JDOS minimum (i.e., suppressed plasmon damping) along the *a*-axis around 0.1 eV, corresponding to the energy range covered in our MIR experiments.

In contrast, the JDOS along the *b*-axis (blue curves, Fig. 4C, D) reveals a series of peaks that are roughly 10 times higher than the *a*-axis JDOS around 0.1 eV. Here, the *b*-axis peaks are composed of both intraband and interband transitions in roughly equal proportion. Since the quality factor scales with the ratio between the plasma frequency and the scattering rate ($Q \sim \frac{{\omega }_{p}}{\gamma }$)^22^ and the *a*- and *b*-axis plasma frequencies are similar (Fig. S3), our calculated damping ratio explains the measured order-of-magnitude reduction of the $Q$ factor along the *b*-axis compared to the *a*-axis for SPPs in the MIR. The notion of an enhanced *b*-axis scattering rate is also consistent with our nano-THz data, since the group velocity of low-momentum THz plasmons is in fact impacted by the scattering rate as described in ref. ^45^. The reduction of the *b*-axis THz group velocity by 30% in Fig. 3C, D is therefore in accord with the scenario of an increased *b*-axis scattering rate. Thus, the totality of our analyses of SPPs in the MIR and THz regimes reveal that anisotropic SPP propagation in graphene/CrSBr is a cooperative effect of strong electronic anisotropy in CrSBr and proximate charge transfer.

## Discussion

We have performed a multi-modal STM and s-SNOM experimental and theoretical study of graphene/CrSBr heterostructures, revealing significant proximity-induced reciprocal charge transfer and electronically-mediated plasmonic anisotropy. By leveraging sensitivity to both the local electronic and nano-optical behavior of graphene/CrSBr, we unravel the subtle interplay of intrinsic optical properties, electronic anisotropy, and emergent plasmonic damping. Combined with theoretical insights provided by first-principles calculations, our observations yield a complete mechanistic picture for engineering uniaxial SPPs in graphene, and enable 2D manipulation of polaritons in an atomically-thin material.

Our results have significant implications for the manipulation of graphene SPPs and 2D polaritons in general, and outline a novel approach for inducing anisotropy in intrinsically isotropic media. Indeed, previously proposed routes for achieving uniaxial or anisotropic SPPs rely on complex device fabrication^17^ or intrinsic anisotropy in the SPP host-medium^47^— significantly limiting the potential material platforms for engineering 1D light. Using our proximity-based approach, it is now possible to use uniaxial graphene SPPs to direct plasmonically-mediated energy-transfer in 2D on a non-volatile platform with nanoscale precision—enabling 2D lensing, waveguiding, and canalization in the monolayer limit. Our heterostructure could thus be integrated into optical circuits to provide space-efficient directional-coupling of photonic elements (e.g., waveguides, resonators, and emitters), acting as both a directional conduit and momentum filter.

The behavior observed in this study is generic to graphene-based charge transfer heterostructures composed of anisotropic semiconducting building blocks (e.g., black phosphorous, ReSe_2_), introducing a new class of quasi-1D plasmonic heterostructures. We also anticipate that this behavior can be further manipulated through modulation of the graphene/CrSBr chemical potential and twist-engineering multiple graphene-CrSBr interfaces. In addition, it is likely that other interlayer effects can be exploited in graphene/CrSBr to tune directional SPP transport, including anisotropic shake-off bands^48^, moiré-induced Brillouin zone folding^43,44^, and quasi-1D charge density wave formation. Finally, we foresee opportunities for electronic and plasmonic manipulation of 2D magnetically-ordered phases in CrSBr using our charge-transfer platform and vice versa.

## Methods

### Device fabrication

Single crystals of CrSBr were synthesized using a chemical vapor transport reaction with source and sink zone temperatures of 930 °C and 850 °C, respectively. Additional details of the CrSBr synthesis can be found in ref. ^49^.

For s-SNOM devices, flakes of few-layer CrSBr, graphene, and few-layer *h*BN (<8 nm) were obtained via mechanical exfoliations. We note that CrSBr naturally cleaves along the principal *a*- and *b*-axes, with a high *a*- to *b*-axis aspect ratio in resulting microcrystals. As such, the relative orientation of graphene plasmon propagation with respect to the underlying CrSBr can be readily identified from the macroscopic features of the stack; this assignment was confirmed with polarized photoluminescence experiments (Fig. S6). Flake thicknesses were identified optically and confirmed with atomic force microscopy (AFM). The *h*BN/graphene/CrSBr heterostructure was prepared with the dry stamp method^50^. A transparent polydimethylsiloxane (PDMS) cube (~ 1 × 1 × 1 mm^3^) was covered by a thin polycarbonate (PC) polymer film and used to pick up the top *h*BN layer. After picking up the graphene layers with the *h*BN, the heterostructure was transferred onto the trilayer CrSBr at elevated temperatures ( ~ 120–180 °C). The sample surface was then washed with chloroform, acetone, and isopropyl alcohol to remove the melted PC polymer. To further remove residual transfer polymer and interfacial bubbles, the surfaces of *h*BN/graphene/CrSBr heterostructures were imaged with approximately 1 nN of contact force using contact-mode AFM with line spacing of < 100 nm.

For STM experiments, exfoliated flakes were instead picked up in the following sequence: *h*BN, CrSBr, then graphene. Polymer-supported stacks were then flipped and deposited onto a Si/SiO_2_ substrate with no further processing. Electrical contact was established to the graphene layer by microsoldering using Field’s metals^51^.

### Scanning tunneling microscopy and spectroscopy

STM/STS measurements were performed in a home-built, ultra-high vacuum system at 5.7 K. Atomically sharp tips were electrochemically etched from tungsten wire and spectroscopically calibrated using Shockley surface states on single crystal Au(111)^52^. Multiple independently prepared tips were used to verify the accuracy and reproducibility of the measurements.

### Scanning Near-field Optical Microscopy

The MIR s-SNOM and KPFM measurements were performed on a commercial Neaspec system under ambient conditions using commercial Arrow^TM^ AFM probes with nominal resonant frequencies of *f* = 75 kHz or 256 kHz. Tunable continuous wave quantum cascade lasers produced by Daylight Solutions were used spanning wavelengths from 9 to 11.7 μm. The detected signal was demodulated at the fourth harmonic of the tip tapping frequency in order to minimize far-field contributions to the scattered light. The fourth harmonic of the near-field scattering amplitude (S_4_) and phase (Φ_4_) were collected simultaneously using a pseudoheterodyne interferometry technique.

The THz s-SNOM measurements were performed on a commercial Neaspec system under ambient conditions using AFM tips produced by Rocky Mountain Nanotechnology, LLC with a nominal resonant frequency of 30– 80 kHz. The THz broadband pulse is generated and detected using a pair of photo-conductive antennas (PCAs, Menlo Systems GmbH). The THz radiation from the PCA emitter is collimated by a TPX lens and focused onto the tip and sample by a parabolic mirror. The scattered field is detected by an unbiased PCA in the time domain using a 50 fs, 780 nm gate beam, and the photocurrent signal is demodulated by a lock-in amplifier at harmonics of the tip tapping frequency.

### Ab-initio calculations of graphene/CrSBr heterostructures

First principles calculations were performed utilizing DFT implemented in the Quantum ESPRESSO package^53^. Norm-conserving pseudopotentials were employed alongside a plane-wave energy cutoff of 90 Ry^54^. For structural relaxation, the spin-polarized Perdew-Burke-Ernzerhof exchange-correlation functional was employed with van der Waals corrections (PBE-D3)^55^. The structures were fully relaxed until the force on each atom was < 0.005 eV/Å. In monolayer CrSBr, the lattice constants along the *a* and *b* axes were determined to be 3.54 Å and 4.72 Å, respectively. In monolayer graphene, the lattice constant *a* was relaxed to 2.465 Å. The graphene/monolayer CrSBr heterostructure was constructed with a supercell of 5 × 2 graphene rectangular conventional cell containing 4 carbon atoms stacked atop a 6 × 1 CrSBr supercell, aligning the *a* and *b* axes of CrSBr along the armchair and zigzag directions of the graphene monolayer, respectively. The graphene monolayer experiences compressive strain of ~0.5% along the armchair direction, and compressive strain of ~4% along the zigzag direction. The vdW spatial gap between the CrSBr layer (from top Br atoms) and the graphene monolayer is 3.46 Å. A vacuum region of 15 Å was added in the out-of-plane direction to avoid interaction between periodic images. Brillouin zone sampling in the graphene/monolayer CrSBr heterostructure was performed using an 8 × 30 × 1 *k*-grid. Dipole correction was applied in all calculations of the graphene/monolayer CrSBr heterostructure^56^. A Gaussian smearing of 1 meV was adopted for electron occupation. The first-principles calculation of JDOS for the estimation of plasmon damping is performed on a 24 × 96 × 1 *k*-grid for the graphene/monolayer CrSBr heterostructure with supercell, and on a 144 × 96 × 1 *k*-grid for the free-standing monolayer CrSBr calculation with doping, so the smallest sampled wavevector for plasmon damping is $2\times {10}^{5}$ cm^–1^ along both *a* and *b* axes. The Brillouin zone unfolding in the JDOS calculation of the graphene/monolayer CrSBr supercell utilizes the BandUp code^57,58^. The RPA dielectric function calculations are performed using the BerkeleyGW code^59^. The inverse of the dielectric function is calculated on a 12 × 48 × 1 *k*-grid, with a cutoff energy of 8.0 Ry, and a slab truncation of the Coulomb interaction. The smallest sampled plasmon wavevector in the dielectric function calculation is $4\times {10}^{5}$ cm^–1^ along both *a* and *b* axes, which is limited by the large computational cost of large supercells. The frequency dependence of the inverse dielectric function is calculated by the Adler-Wiser^60,61^ formalism implemented in the BerkeleyGW code, with an energy broadening of 5 meV. The inverse dielectric function shows the plasmon peaks with linewidths on the order of 20 meV, with a larger linewidth for the plasmon mode along the *b*-axis.

## Supplementary information

Supplementary Information

Transparent Peer Review file

## Data Availability

All raw data presented in the manuscript are available through the Figshare public repository linked to this manuscript.
