# Supplementary material for: Engineering anisotropic electrodynamics at the graphene/CrSBr interface
Source: Nat Commun. 2025 Feb 21;16:1853. doi: 10.1038/s41467-025-56804-y (PMC11845594; doi:10.1038/s41467-025-56804-y)
Supplement: Supplementary file 1 — Supplementary Information [file 41467_2025_56804_MOESM1_ESM.pdf]

Supplementary Information

Engineering anisotropic electrodynamics at the graphene/CrSBr interface

Daniel J. Rizzo[1,*], Eric Seewald[1], Fangzhou Zhao[2], Jordan Cox[3], Kaichen Xie[4], Rocco A. Vitalone[1], Francesco L. Ruta[1,5], Daniel G. Chica[3], Yinming Shao[1,6], Sara Shabani[1], Evan J. Telford[1,3], Matthew C. Strasbourg[7], Thomas P. Darlington[1,7], Suheng Xu[1], Siyuan Qiu[1], Aravind Devarakonda[1,5], Takashi Taniguchi[8], Kenji Watanabe[9], Xiaoyang Zhu[3], P. James Schuck[7], Cory R. Dean[1], Xavier Roy[3,*], Andrew J. Millis[1], Ting Cao[4], Angel Rubio[2,10,11], Abhay N. Pasupathy[1,*], D.N. Basov[1,*]

[1]Department of Physics, Columbia University, New York, NY, 10027, USA

[2]Theory Department, Max Planck Institute for Structure and Dynamics of Matter and Center for Free-Electron Laser Science, 22761 Hamburg, Germany

[3]Department of Chemistry, Columbia University, New York, NY, 10027, USA

[4]Department of Materials Science and Engineering, University of Washington, Seattle, WA 98195, USA

[5]Department of Applied Physics and Applied Mathematics, Columbia University, New York, NY, 10027, USA

[6]Department of Physics, Pennsylvania State University, University Park, Pennsylvania 16802, USA

[7]Department of Mechanical Engineering, Columbia University, New York, NY, 10027, USA

[8]Research Center for Materials Nanoarchitectonics, National Institute for Materials Science, 1-1 Namiki, Tsukuba 305-0044, Japan

[9]Research Center for Electronic and Optical Materials, National Institute for Materials Science, 1-1 Namiki, Tsukuba 305-0044, Japan

[10]Center for Computational Quantum Physics, Flatiron Institute, New York, New York 10010, USA

[11]Nano-Bio Spectroscopy Group, Universidad del País Vasco UPV/EHU, San Sebastián 20018, Spain

*Correspondence to: db3056@columbia.edu, apn2108@columbia.edu, xr2114@columbia.edu and djr2181@columbia.edu

Table of Contents:

**Calculating *Q* for the experimental stack.**	S3
**Ab-initio calculations for graphene/bilayer CrSBr heterostructure.**	S6
**Figure S1. Defect scattering and moiré-dependence of STS on graphene/CrSBr.**	S7
**Figure S2. Kelvin probe force microscopy of CrSBr.**	S8
**Figure S3. Frequency-dependent near-field measurements of graphene/CrSBr.**	S9
**Figure S4. CrSBr FTIR spectra and associated permittivity.**	S10
**Figure S5. DFT supercell and spin-polarized band structures.**	S11
**Figure S6. Polarized photoluminescence of *h*BN/graphene/CrSBr.**	S12
**Figure S7. Background subtraction of THz space-time maps.**	S13
**Table S5. The calculated joint density of state (JDOS) for plasmon damping.**	S14
**References**	S15

Calculating Q for the experimental stack

To calculate the expected Q-factors explicitly, we account for all layers in our van der Waals (vdW) stack using the following approximation:[1]

$$Q^{-1} = \frac{q_2}{q_1} \approx \frac{\sigma_1}{\sigma_2} + \frac{\epsilon_2^{eff}}{\epsilon_1^{eff}} = \frac{\gamma_g(\omega)}{\omega} + \frac{\epsilon_2^{eff}}{\epsilon_1^{eff}} \tag{S1}$$

where $\sigma = \sigma_1 + i\sigma_2$ is the complex optical conductivity of graphene, $\gamma_g(\omega)$ is the frequency-dependent graphene scattering rate,[1] and $\epsilon^{eff} = \epsilon_1^{eff} + i\epsilon_2^{eff}$ is the effective permittivity of the material surrounding graphene. Here, ϵ^{eff} depends on the dielectric functions of the media surrounding graphene (but not graphene itself), and is composed of both substrate and superstrate components. Diagram S1 shows a schematic of our experimental stack and Tables S1–S4 show the associated oscillator parameters for each layer.

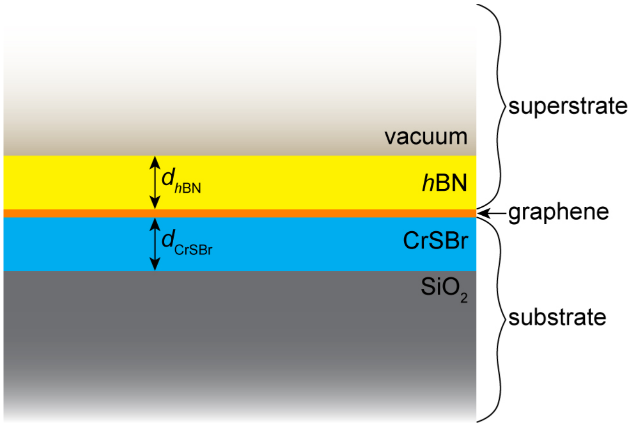

Diagram S1. Schematic of experimental heterostructure used for optical modeling.

Here, the in-plane ($a = xy$) and out-of-plane ($a = z$) responses of hBN were modeled with single-oscillator Drude-Lorentz forms:

$$\epsilon_{hBN}^{a}(\omega) = \epsilon_{\infty}^{a} + \epsilon_{\infty}^{a} \frac{(\omega_{LO}^{a})^2 - (\omega_{TO}^{a})^2}{(\omega_{TO}^{a})^2 - \omega^2 - i\gamma^a\omega} \tag{S2}$$

And the SiO_2 and CrSBr layers were modeled as the sum of multiple Lorentzian oscillators:

$$\epsilon(\omega) = \epsilon_{\infty} + \sum_i \frac{\Omega_i}{\omega_i^2 - \omega^2 - i\gamma_i\omega}$$

(S3)

Where the *a*- and *b*-axis permittivities of CrSBr have different oscillator parameters. The superstrate is composed of a semi-infinite layer of vacuum and an 8.7-nm-thick layer of *h*BN, and the substrate consists of a semi-infinite layer of SiO_2 with a 2.4-nm-thick layer of CrSBr. The effective superstrate permittivity, ϵ_{sup}^{eff} is given by[2]

$$\epsilon_{sup}^{eff} = \epsilon_{hBN}^{z} \frac{\epsilon_0 + \epsilon_{hBN}^{z} \tanh(-ik_{sup} d_{hBN})}{\epsilon_{hBN}^{z} + \epsilon_0 \tanh(-ik_{sup} d_{hBN})}, \quad k_{sup} = i \frac{\sqrt{\epsilon_{hBN}^{xy}}}{\sqrt{\epsilon_{hBN}^{z}}} q .$$

(S4)

Where d_{hBN} is the thickness of the *h*BN layer, ϵ_{hBN}^{xy} and ϵ_{hBN}^{z} are the in-plane and out-of-plane components of the *h*BN permittivity, and $\epsilon_0 = 1$ is the vacuum permittivity. The effective superstrate permittivity, ϵ_{sub}^{eff} can be similarly addressed by substituting $\epsilon_{hBN} \rightarrow \epsilon_{CrSBr}$, $\epsilon_0 \rightarrow \epsilon_{SiO2}$, and $d_{hBN} \rightarrow d_{CrSBr}$:

$$\epsilon_{sub}^{eff} = \epsilon_{CrSBr}^{z} \frac{\epsilon_{SiO2} + \epsilon_{CrSBr}^{z} \tanh(-ik_{sub}^{j} d_{CrSBr})}{\epsilon_{CrSBr}^{z} + \epsilon_{SiO2} \tanh(-ik_{sub}^{j} d_{CrSBr})}, \quad k_{sub}^{j} = i \frac{\sqrt{\epsilon_{CrSBr}^{j}}}{\sqrt{\epsilon_{CrSBr}^{z}}} q .$$

(S5)

Where *j* = *a* or *b* depending on whether we are solving for the effective permittivity along the *a*- or *b*-axis of CrSBr. The overall effective permittivity ϵ^{eff} is the average of the superstrate and substrate permittivities:

$$\epsilon^{eff}(\omega, q) = (\epsilon_{\text{sub}} + \epsilon_{\text{sup}})/2$$

(S6)

For Eq. (S1), we evaluate $\epsilon^{eff}(\omega, q)$ at momenta q_1 given by the energy-moment dispersion extracted from maxima in Im r_p such that $\epsilon^{eff} \equiv \epsilon^{eff}(\omega, q_1)$. The results are plotted in Fig. S3D.

	ω_{TO} (cm^{-1})	ω_{LO} (cm^{-1})	γ (cm^{-1})	ϵ_∞
ϵ^{xy}	1360	1614	7	4.9
ϵ^{z}	760	825	2	2.95

Table S1: Oscillator parameters for *h*BN[3] .

i	ω_i (cm^{-1})	Ω_i (cm^{-1})	γ_i (cm^{-1})
1	1270	224.76i	216
2	1205	208.47	78
3	1072	865.50	49
4	802.3	310.28	80

Table S2: Oscillator parameters for SiO_2. The high-frequency permittivity is $\epsilon_\infty = 1.96$. SiO_2 parameters derived from ref. [4]

i	ω_i (cm^{-1})	Ω_i (cm^{-1})	γ_i (cm^{-1})
1	501.41	2143.41	0
2	771.73	239.80	43.54
3	838.63	928.04	298.45
4	1240.73	2851.03	1195.43
5	2028.01	3413.46	1343.38
6	2881.99	3456.49	1557.61

Table S3: Oscillator parameters for CrSBr *a*-axis. The high-frequency permittivity for the CrSBr *a*-axis is $\epsilon_\infty^a = 14.45$.

i	ω_i (cm^{-1})	Ω_i (cm^{-1})	γ_i (cm^{-1})
1	110.31	3325.26	0
2	676.12	113.59	18.86
3	705.53	325.61	52.77
4	786.22	831.29	201.00
5	1019.84	2043.03	691.51
6	2925.39	3823.45	2692.67
7	1566.03	3081.89	1550.68

Table S4: Oscillator parameters for CrSBr *b*-axis. The high-frequency permittivity for the CrSBr *b*-axis is $\epsilon_\infty^b = 17.72$.

Ab-initio calculations for graphene/bilayer CrSBr heterostructure

First principles calculations were performed utilizing DFT implemented in the Quantum ESPRESSO package.[5] Norm-conserving pseudopotentials were employed alongside a plane-wave energy cutoff of 85 Ry.[6] For structural relaxation, the spin-polarized Perdew-Burke-Ernzerhof exchange-correlation functional was employed with van der Waals corrections (PBE-D2).[7] The structures were fully relaxed until the force on each atom was <0.005 eV/Å. In bilayer CrSBr, the lattice constants along the *a* and *b* axes were determined to be 3.51 Å and 4.71 Å, respectively, and the interlayer distance (Cr-Cr) was calculated to be 8.09 Å. In monolayer graphene, the lattice constant *a* was relaxed to 2.457 Å. The graphene/bilayer CrSBr heterostructure was constructed with an 8 × 2 graphene supercell stacked atop a 5 × 1 CrSBr supercell, aligning the *a* and *b* axes of the CrSBr bilayer along the armchair and zigzag directions of the graphene monolayer, respectively. The graphene monolayer experiences tensile strain of ~3% along the armchair direction, and compressive strain of ~4% along the zigzag direction. The vdW spatial gap between the top CrSBr layer (from the top Br atoms) and the graphene monolayer is 3.38 Å. A vacuum region of 15 Å was added in the out-of-plane direction to avoid interaction between periodic images. Brillouin zone sampling in the graphene/bilayer CrSBr heterostructure was performed using an 8 × 30 × 1 *k*-grid. Dipole correction was applied in all calculations for the graphene/bilayer CrSBr heterostructure.[8] A Gaussian smearing of 1 meV was adopted for electron occupation. We note that the ground state of bilayer CrSBr changes from AFM to FM in our calculations, with a small energy difference < 0.1 meV/(unit cell-layer) between the phases. Calculations using the AFM ground states do not substantially change the amount of charge transferred or the CrSBr effective mass compared with the FM calculations (Fig. S5).

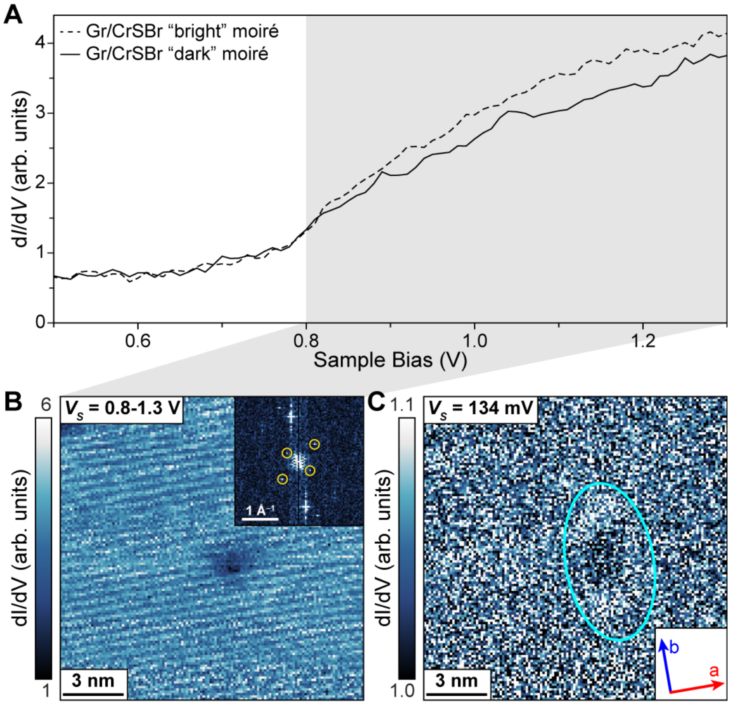

Figure S1. Defect scattering and moiré-dependence of STS on graphene/CrSBr. (**A**) dI/dV point spectra collected at different locations within the second-order moiré pattern formed at the graphene/CrSBr interface. Spectra collected in the "bright" moiré regions (dashed black line) display systematically greater spectral intensity compared to those in "dark" moiré regions (solid black line) over sample biases 0.8 V to 1.3 V. (**B**) dI/dV map showing integrated spectral intensity over sample biases 0.8 V through 1.3 V. A periodic modulation in the spectral intensity commensurate with the second-order moiré pattern is evident. Inset: FFT of the integrated dI/dV map showing Bragg peaks associated with the second-order moiré pattern (yellow circles). (**C**) dI/dV map taken at V_s = 134 mV showing LDOS associated with anisotropic quasiparticle scattering that registers with the underlying a- and b-axes of CrSBr.

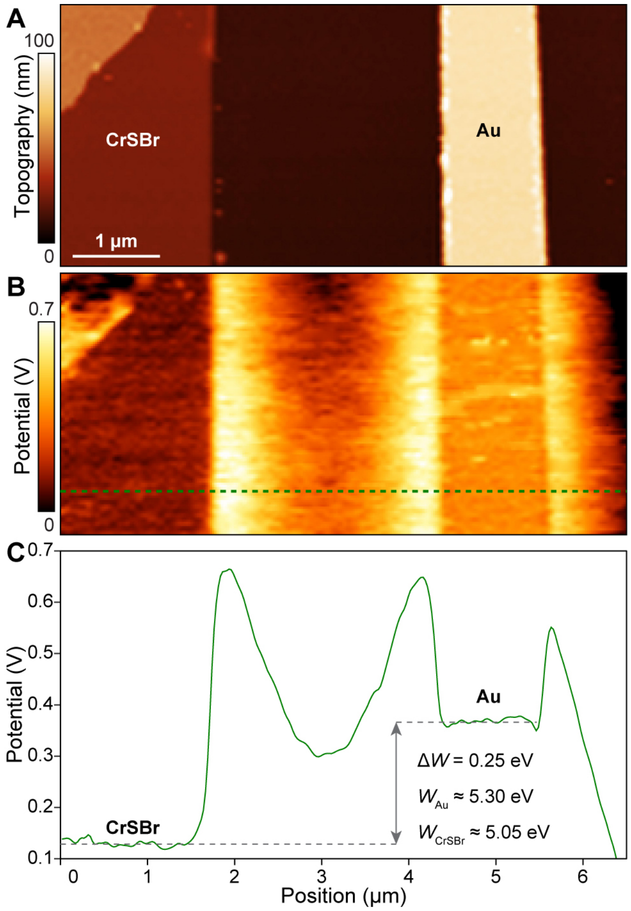

Figure S2. Kelvin probe force microscopy of CrSBr. (**A**) AFM topography of a CrSBr microcrystal next to a Au contact. (**B**) KPFM image of the region shown in panel (A). (**C**) Average linecut of the contact potential measured along the dashed green line in panel (B). The difference in the work functions of Au and CrSBr is measured to be 0.25 eV. Given a nominal value of 5.30 eV for a vacuum-deposited gold thin film,[9] the work function of CrSBr is measured to be 5.05 eV.

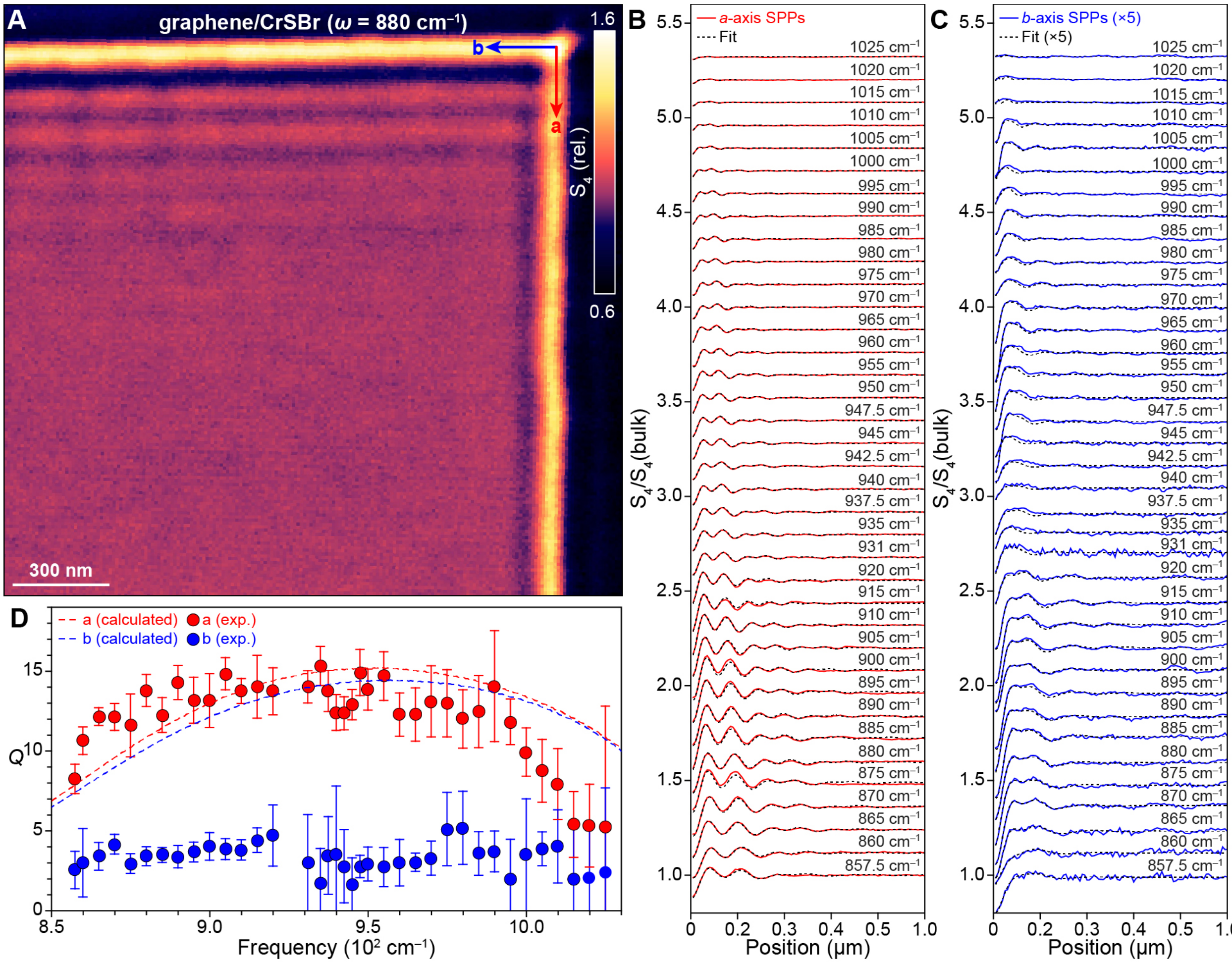

Figure S3. Frequency-dependent near-field measurements of graphene/CrSBr. (**A**) Map of the near-field S_4 amplitude in a graphene/CrSBr heterostructure reproduced from Fig. 2 of the main manuscript ($\omega = 880$ cm^{-1}). (**B**) Average experimental line profile for plasmonic fringes running parallel to the CrSBr *a*-axis for frequencies ranging from $\omega = 857.5$ cm^{-1} to 1025 cm^{-1} (red curves). The values of Q were extracted by fitting the line profiles to the functional ansatz $S_0 + A\frac{e^{-iqx}}{R^a+x^a} + BH_0^{(1)}(2qx)$,[2, 10] (black dashed curves) where S_0 is the bulk near-field amplitude, R is the tip radius (25 nm), $H_0^{(1)}$ is the first Hankel function of order zero, A and B are sample angle- and tip-dependent scaling factors, respectively, and a is a geometric factor ≈0.1. The plane-wave term accounts for light coupled in directly from the edge of the heterostructure that produces near-field oscillations with a period equal to the SPP wavelength (so-called λ-fringes). The Hankel term accounts for cylindrical tip-launched SPPs that interfere upon reflection from the sample edge, leading to near-field oscillations with a period equal to half the SPP wavelength (so-called λ/2-fringes). (**C**) Same as (B) but for fringes running parallel to the CrSBr *b*-axis (blue curves are experiment and black dashed curves are fits). For *b*-axis line profiles with $\omega > 920$ cm^{-1}, q_1 was constrained to be equal to that extracted from the associated *a*-axis profile. (**D**) The experimentally-extracted frequency-dependent Q-factor for SPPs propagating along the *a*-axis (red circles) and *b*-axis (blue circles). Error bars are extracted from the standard error of q_1 and q_2 when fitting the line profiles in (B) and (C). The expected values of Q_a (dashed red curve) and Q_b (dashed blue curve) are plotted based on intrinsic optical parameters (see eq. S1).

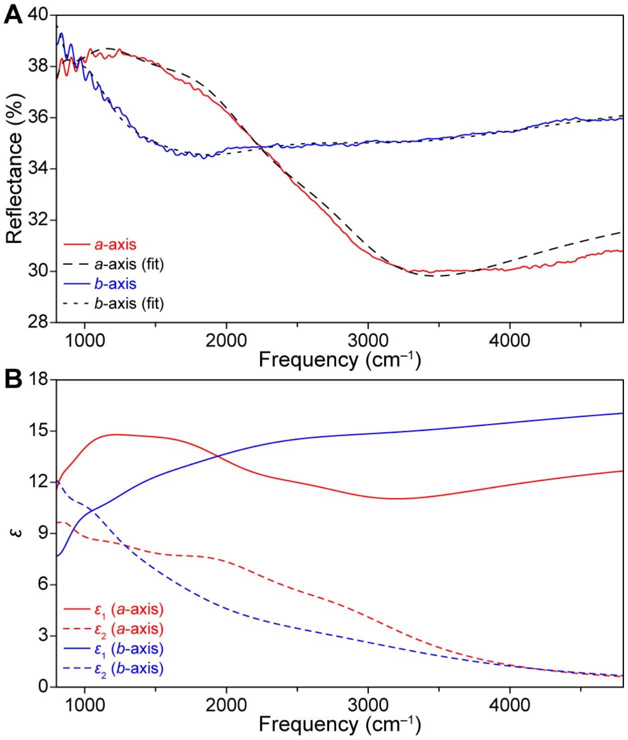

Figure S4. CrSBr FTIR spectra and associated permittivity. (**A**) Far-field FTIR spectra collected on a SiO_2-supported CrSBr microcrystal for light polarized along the *a*-axis (red line) and *b*-axis (blue line). The dashed and dotted black lines show the computed reflectance based on the best fit optical parameters (see Tables S3 and S4). (**B**) The *a*- (red lines) and *b*- (blue lines) components of the real (solid lines) and imaginary (dashed lines) permittivity extracted from the FTIR spectra in panel (A) (see Tables S3 and S4).

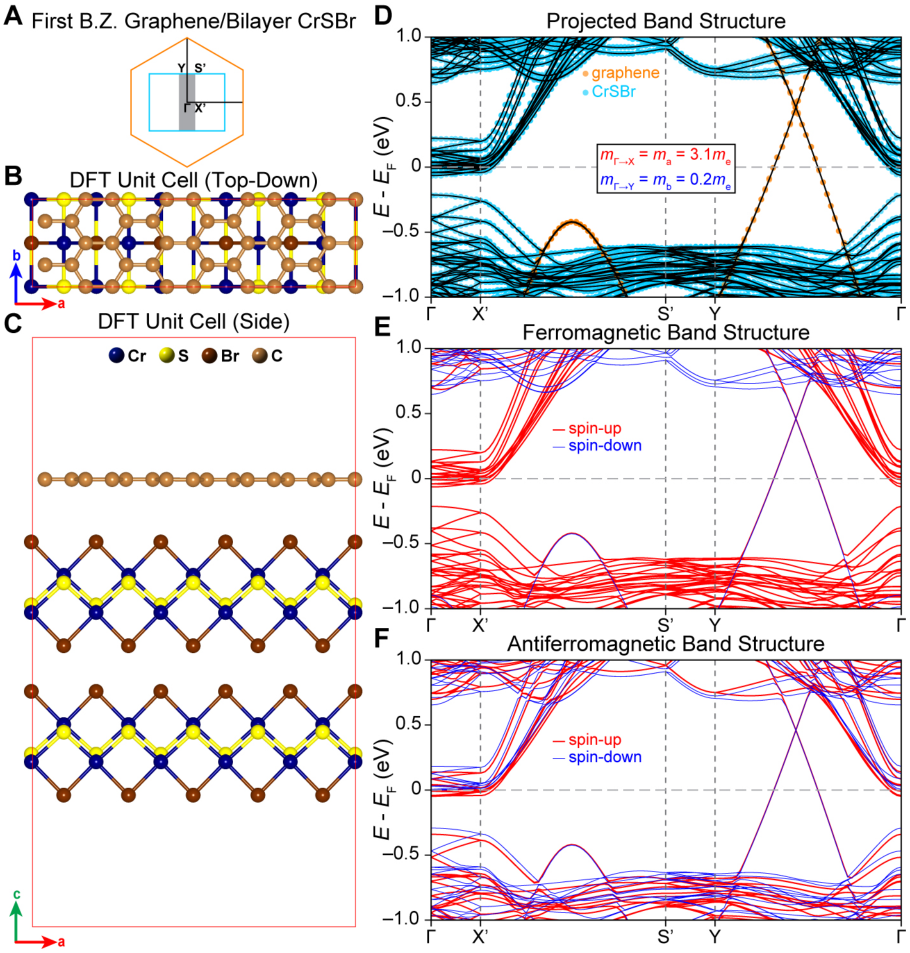

Figure S5. DFT supercell and spin-polarized band structures. (**A**) Schematic of the first Brillouin zone of graphene (orange hexagon), of CrSBr (cyan rectangle), and of the supercell shown in (B) and (C) (solid gray rectangle). High symmetry points associated with the isolated layers and the composite structure are labelled. (**B**) Top-down and (**C**) side views of the DFT supercell used for calculations in panels (D) – (F). The red line indicates the boundaries of the supercell. (**D**) Band structure for the graphene/bilayer CrSBr supercell shown in (B) and (C). States localized in the graphene (CrSBr) layer are indicated with orange (cyan) circles. A shift of ~0.5 eV in E_{Dirac} is observed for the hole-doped graphene layer. The CrSBr conduction band is observed to be electron-doped, with *a*-axis carriers possessing an order-of-magnitude greater effective mass ($m_{\Gamma\rightarrow X} = m_a = 3.1m_e$) than *b*-axis carriers ($m_{\Gamma\rightarrow Y} = m_b = 0.2m_e$). (**E**) Spin-up (red lines) and spin-down (blue) band structure for the supercell shown in panels (B) and (C) given interlayer ferromagnetic ordering in the CrSBr bilayer. (**F**) Same as (E) but for interlayer antiferromagnetic ordering in the CrSBr bilayer.

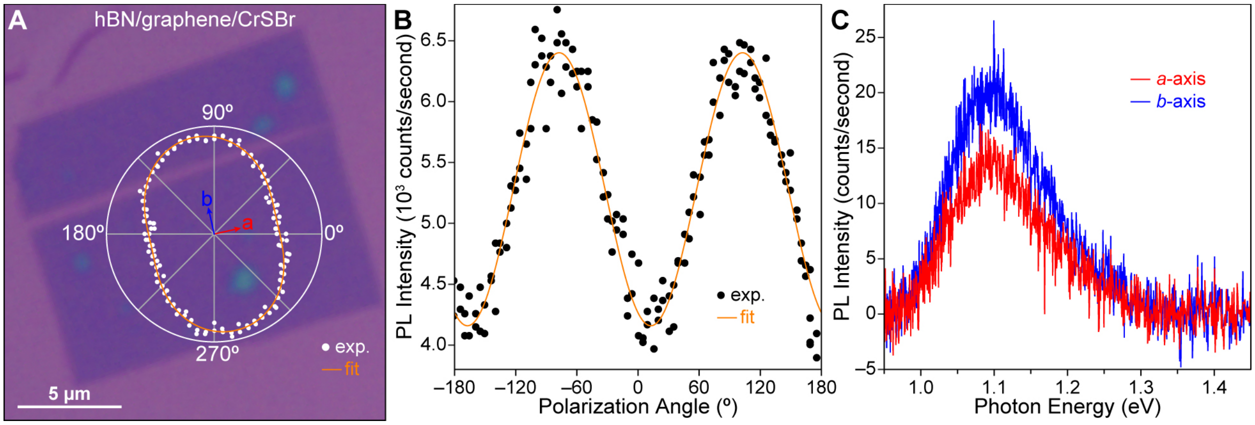

Figure S6. Polarized photoluminescence of *h*BN/graphene/CrSBr. (**A**) Optical overview of vdW heterostructure consisting of *h*BN/graphene/CrSBr. The CrSBr microcrystal naturally exfoliates such that the *a*-to-*b*-axis aspect ratio is greater than one. A polar plot of the PL intensity is overlaid on the CrSBr microcrystal, indicating that the PL intensity is at a maximum along the *b*-axis and minimum along the *a*-axis. (**B**) Plot of the angle-dependent PL intensity reproduced from panel (A). (**C**) Characteristic PL spectra taken along the CrSBr *a*- and *b*-axes. Compared to PL in isolated few-layer CrSBr,[11] the PL spectrum is red-shifted by ~200 meV and presents with less angular anisotropy.

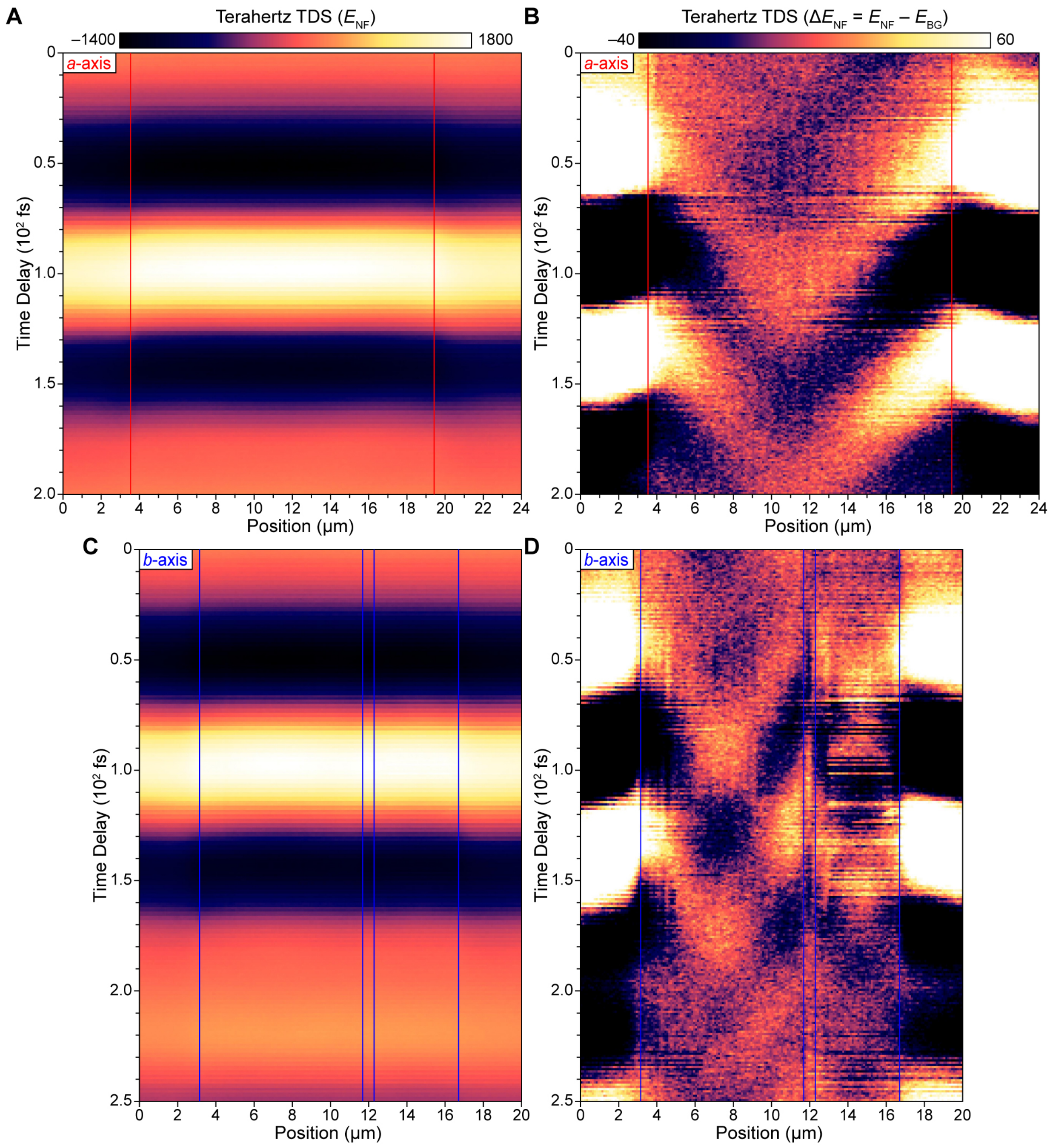

Figure S7. Background subtraction of THz space-time maps. (**A**) Raw space-time map of the near-field electric field E_{NF} collected along the a-axis of a graphene/CrSBr heterostructure. The signal is dominated by the waveform of the THz pulse. (**B**) The space-time map shown in (A) with the background THz pulse removed, leaving just the near-field plasmon electric field ($\Delta E_{NF} = E_{NF} - E_{BG}$). Here, each line is leveled by subtracting the mean signal within the CrSBr flake (CrSBr edges indicated by the red vertical lines). (**C**) Same as (A) but conducted along the CrSBr b-axis. (**D**) Same as (B) but collected along the CrSBr b-axis. We note the presence of a crack in the middle of the flake, giving rise to plasmonic reflection at four boundaries instead two (indicated by blue vertical lines).

(A)

$\boldsymbol{q_a}$ *or* $\boldsymbol{q_b}$ (**unit**: $\times$ $\mathbf{10^5}\boldsymbol{cm^{-1}}$)	**2**	**4**	**6**	**8**
$\boldsymbol{\Gamma(E = 0.1\,eV,\, q = q_a)}$ (unit: states/eV/Å^2)	0.00323	0.00314	0.00310	0.00306
$\boldsymbol{\Gamma(E = 0.1\,eV,\, q = q_b)}$ (unit: states/eV/Å^2)	0.00258	0.00591	0.02860	0.04527

(B)

$\boldsymbol{q_a}$ *or* $\boldsymbol{q_b}$ (**unit**: $\times$ $\mathbf{10^5}\boldsymbol{cm^{-1}}$)	**2**	**4**	**6**	8
$\boldsymbol{\Gamma(E = 0.1\,eV,\, \lvert q \rvert = q_a)}$ (unit: states/eV/Å^2)	0.00153	0.00220	0.00288	0.00326
$\boldsymbol{\Gamma(E = 0.1\,eV,\, \lvert q \rvert = q_b)}$ (unit: states/eV/Å^2)	0.00969	0.01326	0.01971	0.02505

Table S5. The calculated joint density of state (JDOS) for plasmon damping. (A) The calculated JDOS for plasmon damping for free-standing monolayer CrSBr with the Fermi level shifted due to charge transfer between the interfacial CrSBr layer and graphene (As shown in Fig. 4A of main manuscript). **(B)** The calculated JDOS for plasmon damping for the graphene/monolayer CrSBr heterostructure. All the calculated quantities are integrated over a 0.02 eV energy window centered at 0.1 eV.

References

1. Ni, G.X. *et al.* Fundamental limits to graphene plasmonics. *Nature* **557**, 530-533 (2018).
2. Rizzo, D.J. *et al.* Charge-Transfer Plasmon Polaritons at Graphene/α-$RuCl_3$ Interfaces. *Nano Letters* **20**, 8438-8445 (2020).
3. Caldwell, J.D. *et al.* Sub-diffractional volume-confined polaritons in the natural hyperbolic material hexagonal boron nitride. *Nature Communications* **5**, 5221 (2014).
4. Fei, Z. *et al.* Infrared Nanoscopy of Dirac Plasmons at the Graphene–SiO_2 Interface. *Nano Letters* **11**, 4701-4705 (2011).
5. Giannozzi, P. *et al.* QUANTUM ESPRESSO: a modular and open-source software project for quantum simulations of materials. *Journal of Physics: Condensed Matter* **21**, 395502 (2009).
6. Hamann, D.R. Optimized norm-conserving Vanderbilt pseudopotentials. *Physical Review B* **88**, 085117 (2013).
7. Grimme, S. Semiempirical GGA-type density functional constructed with a long-range dispersion correction. *Journal of Computational Chemistry* **27**, 1787-1799 (2006).
8. Bengtsson, L. Dipole correction for surface supercell calculations. *Physical Review B* **59**, 12301-12304 (1999).
9. Sachtler, W.M.H., Dorgelo, G.J.H. & Holscher, A.A. The work function of gold. *Surface Science* **5**, 221-229 (1966).
10. Woessner, A. *et al.* Highly confined low-loss plasmons in graphene–boron nitride heterostructures. *Nature Materials* **14**, 421-425 (2015).
11. Wilson, N.P. *et al.* Interlayer electronic coupling on demand in a 2D magnetic semiconductor. *Nature Materials* **20**, 1657-1662 (2021).
